# Cellular concepts cure cancer

**DOI:** 10.1007/s00709-023-01841-x

**Published:** 2023-02-08

**Authors:** Peter Nick

**Affiliations:** Botanical Institute, Karlsruhe Institute of Technology, Karlsruhe, Germany

Healing always has been one of the most powerful drivers for the development of science, which holds true for different civilisations. For instance, the *Divine Farmers Plant Science* (*Shénnóng Běncǎojīng*) which was written around 500 BC, not only describes several hundreds of plant drugs that are still in use today in China, but also develops a theoretical framework to explain their effect upon the human organism. Likewise, Dioskurides lists more than 800 medicinal plants and explains their activity in the conceptual framework put down by Galenos. Both examples show that the science of healing, from its very beginnings, fostered a universalistic component transcending the border between humans and plants and, thus, helped to develop biology from a science describing numerous particularities into an analytical science explaining the general principles of living beings. Four contributions to the current issue pick up this impulse of addressing healing in an analytical manner. They do this from different and complementary perspectives.

The work by Shaglouf et al. (2023) develops a multifaceted molecular and cellular scenario explaining, how liver cancers arise, focussing on hepatocyte carcinomas as the most frequent form of liver cancer. Since this disease occurs preferentially in patients suffering from cirrhosis, it remains undetected in the vast majority of cases, which means that it is already incurable, when it becomes detected. Sensitive biomarkers that allow for an early diagnosis are mandatory, therefore. Authors had developed a rat model, where this type of carcinoma can be chemically induced (Shaglouf et al. 2020). In their contribution to the current issue, they use a temporally resolved proteomics strategy comparing mock-treated and cancer-induced animals and validate differentially expressed transcript. For the confirmed data, they compare also biopsy samples from patients diagnosed for this disease. They can pinpoint the pre-mRNA splicing factor 1 homolog as key regulator and can link this with interaction partners, some of which had been already detected in their preceding study (Shaglouf et al. 2020). The congruence in regulation between their rat model and tissue samples from cancer patients show that EGFR and MAPK signalling, as well as splicing are affected during an early phase of cancerogenesis, which allows to define biomarkers for diagnosis that are not only based on correlation, but can be also functionally linked with the disease to be diagnosed.

For the contribution by Pandey et al. (2023), we need to shift perspective and look into a medicinal plant that accumulates liver-protective compounds. The rare medicinal plant *Picrorhiza kurroa* from the Himalaya accumulates picrosides, monoterpenes that can cure cholestasis, the unrestrained accumulation of bile acids, one of the causes for cirrhosis. These precious compounds are enriched in the rhizomes of these plants belonging to the Plantaginaceae. However, they are synthetised in shoots and roots and, therefore, must be transported to their final destination. Secondary metabolites are often mobilised through ATP-binding cassette (ABC) transporters, and the authors use organ-specific transcriptome analysis to find the transporter responsible for picroside repartitioning, which is far from trivial in such an exotic species, where, unlike for the usual model plants, no molecular information is available. From almost 100 candidates (the ABC-transporters are organised in large and diverse gene families); they use a combination of expression analysis by real-time RT-qPCR and comparisons between genotypes differing in the accumulation of picrosides in the young rhizome to cook down complexity to three main transporters in the shoot, two in the root and two in the final destination, the rhizome. This pioneering work allows now not only to understand the dynamics of these metabolites, but also to use these transporters as candidates for breeding new genotypes with elevated levels of these hepatoprotective compounds.

The contributions by Raorane et al. (2023) and Lemos Cruz et al. (2023) address the biosynthesis of not only vincristines, alkaloids with strong activity against leukaemia and lymphomas, but also cancers that are hard to target, such as neuroblastomas. The synthesis of these compounds is very complex and comprises around 50 enzymatic steps that are distributed over different tissues and cell types until the end product is collected in so-called idioblasts in the leaves. Immense amounts of plant material are needed to get sufficient product, such that the price is very high (around 1000 $/g). To develop biotechnological alternatives, this pathway, its localisation and its regulation have been intensively investigated. The long-term goals are biotechnological approaches for its production, for instance by reconstituting the pathway in cell factories. The work by Lemos Cruz et al. (2023) describes now that silencing a key enzyme, a methyl transferase converting the precursor 16-hydroxytabersonine, has only a mild effect on product accumulation leading these authors to ask, whether there are unknown players adopting this task. Their search leads them to a second enzyme. This previously unknown isoform turns out to accept flavonoid substrates, but 16-hydroxytabersonine as well. Using fluorescent assays, such as bimodal fluorescence complementation, they address the possibility that these enzymes form a complex, but this can be ruled out. Instead, both isoforms act as homo-dimers in the cytoplasm. Overexpression of the second isotype does not lead to increases in the downstream product vindoline, such that the biological function of this second isotype seems to reside mainly in flavonoid methylation. Authors come up with a model, where a gene duplication of a flavonoid methylase with broad substrate specificity enabled, via a neofunctionalisation, the synthesis of *Vinca* alkaloids. This work further supports the notion of generic enzymes as drivers of Vinca alkaloid synthesis extending earlier findings of a multifunctional peroxidase generating a-3′,4′-anhydrovinblastine (Sottomayor and Ros Barceló 2003).

Plant cell fermentation using *Catharanthus* cells might be an alternative to the cumbersome extraction from leaf tissue. However, this approach has not been successful, so far, which might be linked with the fact that it seems difficult till impossible to mimick the complex interactions between different cell types with different and complementary metabolic potencies (often termed as competence) in the leaf using a suspension culture of a single cell type. This consideration was the starting point for the work by Raorane et al. (2023) addressing the competence of suspension cell strains of *Catharanthus*. Two strains with complementary metabolic competence were isolated from mature embryos and differed not only in morphology, but also in subcellular details, for instance mitochondrial shape. One of these strains displays traits of idioblasts i.e. the cell type, where the final product accumulates. In a previous work, combination of both strains using a microfluidic chip had allowed to generate the precursor vindoline (Finkbeiner et al. 2022). Elicitating the idioblast-like strain by methyl jasmonate and feeding of the precursor vindoline, vincristine became detectable, albeit only to trace amounts.

All the four contributions address different facets of curing cancers, and, thus, have a certain impact on application. It is possible to develop applications just by high-throughput screening, robotics, automisation and optimisation using Artificial Intelligence, which seemingly renders an analytical understanding of the phenomenon dispensible. However, this would shift science into the realm of mere engineering, and such “unbiased” approaches are not parsimonious with respect to resources (including human labour). Applications that are based on a scientific understanding of the process, one is going to exploit, are far more efficient. In other words: there is no real alternative to hypothesis-driven application.
